# Pea genomic selection for Italian environments

**DOI:** 10.1186/s12864-019-5920-x

**Published:** 2019-07-22

**Authors:** Paolo Annicchiarico, Nelson Nazzicari, Luciano Pecetti, Massimo Romani, Luigi Russi

**Affiliations:** 1 Council for Agricultural Research and Economics (CREA), Research Center for Animal Production and Aquaculture, viale Piacenza 29, 26900 Lodi, Italy; 2 Department of Agricultural, Food and Environmental Science, University of Perugia, Borgo XX Giugno 74, 06121 Perugia, Italy

**Keywords:** Breeding value, Cross-population prediction, Genotyping-by-sequencing, Genotype × environment interaction, *Pisum sativum*, Predictive ability, Yield

## Abstract

**Background:**

A thorough verification of the ability of genomic selection (GS) to predict estimated breeding values for pea (*Pisum sativum* L.) grain yield is pending. Prediction for different environments (inter-environment prediction) has key importance when breeding for target environments featuring high genotype × environment interaction (GEI). The interest of GS would increase if it could display acceptable prediction accuracies in different environments also for germplasm that was not used in model training (inter-population prediction).

**Results:**

Some 306 genotypes belonging to three connected RIL populations derived from paired crosses between elite cultivars were genotyped through genotyping-by-sequencing and phenotyped for grain yield, onset of flowering, lodging susceptibility, seed weight and winter plant survival in three autumn-sown environments of northern or central Italy. The large GEI for grain yield and its pattern (implying larger variation across years than sites mainly due to year-to-year variability for low winter temperatures) encouraged the breeding for wide adaptation. Wider within-population than between-population variation was observed for nearly all traits, supporting GS application to many lines of relatively few elite RIL populations. Bayesian Lasso without structure imputation and 1% maximum genotype missing rate (including 6058 polymorphic SNP markers) was selected for GS modelling after assessing different GS models and data configurations. On average, inter-environment predictive ability using intra-population predictions reached 0.30 for yield, 0.65 for onset of flowering, 0.64 for seed weight, and 0.28 for lodging susceptibility. Using inter-population instead of intra-population predictions reduced the inter-environment predictive ability to 0.19 for grain yield, 0.40 for onset of flowering, 0.28 for seed weight, and 0.22 for lodging susceptibility. A comparison of GS vs phenotypic selection (PS) based on predicted genetic gains per unit time for same selection costs suggested greater efficiency of GS for all traits under various selection scenarios. For yield, the advantage in predicted efficiency of GS over PS was at least 80% using intra-population predictions and 20% using inter-population predictions. A genome-wide association study confirmed the highly polygenic control of most traits.

**Conclusions:**

Genome-enabled predictions can increase the efficiency of pea line selection for wide adaptation to Italian environments relative to phenotypic selection.

**Electronic supplementary material:**

The online version of this article (10.1186/s12864-019-5920-x) contains supplementary material, which is available to authorized users.

## Background

Greater cultivation of grain legumes is a priority for European agriculture to increase its sustainability in terms of soil fertility, energy efficiency, greenhouse gas emissions and crop diversity on the one hand [1–3], and to decrease its huge dependency from international markets for high-protein feedstuff on the other [4]. Grain legumes were grown over just 1.5% of the arable land in Europe in 2014 compared with 14.5% on a worldwide basis [3]. Economically insufficient yield compared to alternative crops is recognized as the main factor that limits grain legume cultivation in Europe [5]. Plant breeding has unanimously been indicated as the main avenue to increase grain legume yields in this region [6, 7].

Field pea (*Pisum sativum* L.) is the most-grown grain legume in Europe, where it displays higher yield potential than other cool-season grain legumes in southern [8] and western Europe [9]. High protein and energy value for animal nutrition [8], and remarkable flexibility of utilization (as grain, hay, or silage) [10], are further assets of this crop. Pea exhibited a relatively high rate of genetic yield gain, estimated as 1.3% per year according to international cultivars evaluated in Italy between 1992 and 2001 [11], and about 2% per year in Canada based on varieties released between 1993 and 2012 [12]. To secure high and stable yield, however, pea breeding has to tackle several abiotic and biotic stresses, most of which have specific regional relevance [12]. Terminal drought is the main stress limiting pea yield in Mediterranean-climate regions, and escape via early phenology is a key trait to cope with it, although an intrinsic stress tolerance can be present in pea germplasm [13]. Three phenological types are recognised in pea, namely, the European spring and winter types and the Mediterranean type [14], the third type being adapted to autumn sowing in mild-winter areas, whereas winter and spring types differ for winter hardiness and sowing time in central and western Europe. Tolerance to low winter temperatures is a major breeding goal to increase crop yields by shifting from spring sowing to autumn sowing [15, 16], thereby exploiting the longer crop cycle and reducing the terminal stress via earlier crop maturity.

Poor standing ability is a recognised hindrance of pea, and greater tolerance to lodging has been pursued by selecting semi-leafless varieties carrying the *afila* gene (which causes the replacement of leaflets by tendrils). Semi-leafless varieties are less prone to lodging than leafed germplasm, but display variation for standing ability [17, 18]. A reduction of plant stature induced by the introduction of dwarfing genes may further increase the crop standing ability. Variation in plant height is present in semi-dwarf germplasm, though, owing to different combinations of dwarfing genes [19].

Despite the array of breeding targets to aim for, the scarcity of resources devoted to crop improvement relative to those available for major non-legume crops remains a crucial limitation for pea (and other grain legumes), highlighting the importance of devising more efficient selection procedures. So far, the paucity of genomic resources and the need to cope with large genotype × environment interaction (GEI) have been major hindrances to the application of marker-based selection in grain legumes [20]. However, some early studies revealed useful molecular markers linked to pea yield traits [21–23] or pea tolerance to major abiotic or biotic stresses [24, 25]. New opportunities have arisen from the recent development of powerful pea SNP array platforms [26] and genotyping-by-sequencing (GBS) [27, 28], whose cost per SNP data point is lower than array platforms albeit with possibly large amounts of missing data. Besides providing more solid ground for the detection of useful markers [29], these developments have allowed to explore the potential value of genomic selection (GS) as an alternative or, at least, a complement to field-based phenotypic selection (PS). One pioneer study based on SNP array data revealed a good ability to predict two seed yield components and flowering time in a pea germplasm collection [30, 31]. A second study based on GBS data revealed good GS predictive ability for pea grain yield in a severely drought-prone environment, where the correlation between observed and predicted phenotypes in a cross-validation scheme exceeded 0.5 for each of three recombinant inbred line (RIL) populations [13]. A preliminary study for Italian agricultural environments indicated an average predictive accuracy of 0.59 for grain yield of the same RIL populations [32]. Various factors, such as the GS model [33] and the allowed missing rate, could affect the GS predictive ability in these studies [13, 30].

A thorough verification of the value of GS to predict estimated breeding values for pea grain yield requires to take into account GEI effects, which may be large across autumn-sown environments of southern Europe [34–36]. Particularly for Italy, GEI proved to be largely affected by year-to-year variation for timing and extent of winter cold stress, whose effect can be highly damaging in specific years both in subcontinental-climate and Mediterranean-climate areas [15]. This GEI pattern, which implies relatively low genotype × location interaction compared to genotype × year and genotype × location × year interactions, suggested to breed pea for wide adaptation to Italian environments, in contrasts with results for other cool-season grain legumes that support the selection for specific adaptation to subcontinental-climate or to Mediterranean-climate regions (e.g., faba bean [37] and white lupin [38]). The comparison of inter-environment GS-based predictions (i.e., the ability of a GS model to predict pea genotype yield responses in independent environments/years) with inter-environment predictions based on phenotypic data has high practical importance in this context, particularly when expressing its results in terms of yield gain per unit time expected from each selection strategy [39].

Another issue of great practical interest is the ability of a GS model to predict yield responses in germplasm/reference populations different from the population for which the model was defined. Transferability of models would obviously decrease the cost of model development, and would impact the strategies of GS implementation in breeding programs. Inter-population (alias cross-population) predictions in genetically unrelated populations were nil for grain yield in a wheat study [40], and moderate for biomass yield in a study on alfalfa [41]. In pea, inter-population predictions for grain yield varied largely for pairs of RIL populations that shared one common parent [13].

The main objective of this study was to assess the predictive ability of GS for grain yield and a few key agronomic traits of pea across organically-managed or conventionally-managed Italian environments that differed for climatic area and/or cropping year, taking into account inter-environment and inter-population predictions and comparing GS vs PS in terms of expected genetic gain per unit time. GS was previously optimized by assessing different models and data configurations. An additional objective of this study was to investigate GEI patterns for a large set of breeding lines, the phenotypic variation between and within RIL populations, and their implications for pea breeding targeted to Italian environments.

## Results

### Phenotypic variation, genotype × environment interaction and trait interrelationships

In Lodi, the year 2014–15 (with conventional crop management) displayed lower mean grain yield than 2013–14 (with organic management) (Table 1), owing to much higher winter plant mortality (about 27% vs 1%) arising from distinctly colder winter (with absolute minimum temperature of − 11.6 °C vs − 5.7 °C) and to lower rainfall (Additional file 1: Table S1). Perugia, featuring mild winter and moderate rainfall (Additional file 1: Table S1), was the lowest-yielding environment (Table 1), partly because of severe weed competition arising from the organic management.

**Table 1:** Mean trait value of three pea test environments

Trait	Lodi 2013–14	Lodi 2014–15	Perugia 2013–14
Grain yield (t/ha)	6.307 a	4.586 b	2.905 c
Onset of flowering (dd from Apr. 1)	12.42 b	15.36 a	12.44 b
Lodging susceptibility (score 1 = min, 5 = max)	2.50 b	3.67 a	2.18 b
Individual seed weight (g)	0.213 a	0.187 b	0.190 b
Winter plant survival (proportion)	0.991 a	0.726 b	0.995 a

Row means with different letter differ at *P* < 0.05

Results for winter plant survival are reported hereafter only for the environment displaying genetic variation for this trait, i.e., Lodi 2014–15. The RIL population K × A exhibited much greater winter mortality and lower grain yield in this environment, while the three populations did not differ widely for grain yield in the two mild-winter environments (Table 2). The mean responses of the RIL populations reflected well those of their parent lines (Additional file 2: Table S2). The population A × I showed consistently earlier onset of flowering than the other populations (Table 2), whereas the cold-susceptible population K × A was latest-flowering in the cold-winter environment because its surviving plants frequently flowered by means of secondary stems after the winter-killing of their primary stem.

**Table 2:** Mean value, and genetic coefficient of variation (CV_g_), for trait values in three test environments of three pea RIL populations derived from three connected crosses (A × I, 102 lines; K × A, 100 lines; K × I, 104 lines)

		Mean value^c^						CV_g_ (%)^d^					
Trait^a^	Environment^b^	A × I		K × A		K × I		A × I		K × A		K × I	
GY (t/ha)	Lo14	5.994	a	6.326	a	6.541	a	10.1	**	17.5	**	18.2	**
GY (t/ha)	Lo15	5.805	a	2.522	b	5.777	a	28.0	**	51.3	**	33.0	**
GY (t/ha)	Pg14	2.615	b	2.773	b	3.310	a	24.8	**	20.7	**	14.8	**
OF (dd from Apr. 1)	Lo14	10.26	c	13.84	a	13.14	b	18.5	**	37.5	**	33.2	**
OF (dd from Apr. 1)	Lo15	12.45	c	18.72	a	14.81	b	14.2	**	28.3	**	27.7	**
OF (dd from Apr. 1)	Pg14	10.58	b	13.43	a	13.17	a	28.3	**	39.5	**	41.8	**
LS (score 1 = min, 5 = max)	Lo14	2.53	a	2.43	a	2.58	a	20.9	**	9.7	NS	22.9	**
LS (score 1 = min, 5 = max)	Lo15	3.66	b	3.52	ab	3.80	a	7.4	**	0.0	NS	8.7	**
LS (score 1 = min, 5 = max)	Pg14	2.37	a	2.14	a	2.02	a	16.2	**	13.7	NS	23.0	**
SW (g)	Lo14	0.207	b	0.230	a	0.201	b	9.6	**	8.0	**	9.3	**
SW (g)	Lo15	0.190	a	0.192	a	0.180	b	8.2	**	5.8	**	7.9	**
SW (g)	Pg14	0.183	b	0.207	a	0.181	b	9.4	**	8.0	**	9.9	**
WS (proportion)	Lo15	0.877	a	0.436	b	0.865	a	10.8	**	32.2	**	12.8	**

^a^ GY, grain yield; OF, onset of flowering; LS, lodging susceptibility; SW, individual seed weight; WS, winter plant survival

^b^ Lo14, Lodi 2013–14; Lo15, Lodi 2014–15; Pg14, Perugia 2013–14

^c^ Row means followed by different letter differ at *P* < 0.05

^d^ NS and **: genetic variance not different from zero at *P* < 0.05 and different from zero at *P* < 0.01, respectively

Information on within-population genetic variation expressed as genetic coefficient of variation (CV_g_) is reported in Table 2. Variation was found for all traits in all RIL populations except for lodging susceptibility in K × A, which failed to display significant variation in any environment. Therefore, this population was excluded from the assessment of genomic predictions for this trait. The cold-prone environment tended to widen the within-population genetic variation for grain yield and to reduce the variation for lodging susceptibility (Table 2). In this environment, the population K × A combined the greatest cold susceptibility with the largest genetic variation for this trait (Table 2). The three populations displayed comparable within-population variation for the remaining trait-environment combinations, except for a consistent trend towards lower variation of A × I for onset of flowering (Table 2) that was expected from the similar phenology of its parent lines (Additional file 2: Table S2).

Variance component estimation for grain yield of the whole set of RILs indicated that purely genotypic effects (*S*_*G*_^*2*^) were much smaller than GEI effects (*S*_*GE*_^*2*^) (Table 3). In contrast, GEI effects were smaller than purely genotypic effects for lodging susceptibility, and particularly small for onset of flowering and seed weight (Table 3). Estimates of broad-sense heritability on a genotype mean basis across environments averaged across the individual RIL populations indicated only moderate heritability for grain yield (*H*^*2*^ = 0.50) and susceptibility to lodging (*H*^*2*^ = 0.55), and high heritability for onset of flowering and seed weight (*H*^*2*^ = 0.91).

**Table 3:** Estimated components of variance relative to genotype (*S*_*G*_^*2*^) and genotype × environment interaction (*S*_*GE*_^*2*^) and to RIL population (*S*_*R*_^*2*^), genotype within RIL population (*S*_*G(R)*_^*2*^), RIL population × environment interaction (*S*_*RE*_^*2*^) and genotype within RIL population × environment interaction (*S*_*G(R)E*_^*2*^), for traits of 306 pea lines belonging to three RIL populations tested in three environments (Lodi 2013–14; Lodi 2014–15; Perugia 2013–14)

	Analysis without RIL population factor^b^					Analysis with RIL population factor^b^							
Trait^a^	*S*_*G*_^*2*^		*S*_*GE*_^*2*^		*S*_*G*_^*2*^/*S*_*GE*_^*2*^ ratio	*S*_*R*_^*2*^		*S*_*G(R)*_^*2*^		*S*_*RE*_^*2*^		*S*_*G(R)E*_^*2*^	
GY (t/ha)^2^	0.567	**	1.443	**	0.39	0.078	**	0.517	**	1.122	**	0.689	**
OF (dd from Apr. 1)^2^	18.88	**	2.73	**	6.91	3.98	**	16.30	**	1.18	**	1.96	**
LS [score (1 = min, 5 = max)]^2^	0.113	**	0.042	**	2.68	0.000	NS	0.123	**	0.015	**	0.012	NS
SW [(g)^2^ × 1000]	0.333	**	0.056	**	5.94	0.118	**	0.254	**	0.043	**	0.030	**
WS (proportion)^2^	–	–	–	–	–	0.063	**	0.014	**	–	–	–	–

^a^ GY, grain yield; OF, onset of flowering; LS, lodging susceptibility; SW, individual seed weight; WS, winter plant survival. All traits assessed in three environments except winter plant survival, assessed only in Lodi 2014–15

^b^ NS and **: component of variance not different from zero at *P* < 0.05 and different from zero at *P* < 0.01, respectively

Variance component estimation in the presence of the RIL population factor indicated that the genotypic variation between RIL populations was definitely smaller than the average within-population variation, except for winter plant survival (Table 3). The variation of population × environment interaction tended to be higher for grain yield, and roughly comparable for the other traits, relative to the average GEI variation within populations (Table 3). For lodging susceptibility, the variation between RIL populations across environments and the average within-population variation for GEI were not significant (Table 3).

Genetic correlation values for line responses were lower across years in Lodi than across locations in the same year for all traits (Table 4), indicating larger GEI across years than across sites. The consistency of genotype responses was particularly low for grain yield across years in Lodi (*r*_*g*_ = 0.325), while being very high for lodging susceptibility across sites in 2013–14 (*r*_*g*_ near unity, non-significant GEI; Table 4).

**Table 4:** Significance and extent (as genetic correlation *r*_*g*_ for genotype response across environments) of genotype × environment interaction across pairs of test environments, for traits of 306 pea lines belonging to three RIL populations

	Years 2013–14 vs 2014–15 in Lodi^a^		Lodi vs Perugia in 2013-14^a^	
Trait	*P* value	*r*_*g*_	*P* value	*r*_*g*_
Grain yield	**	0.325	**	0.822
Onset of flowering	**	0.889	**	0.955
Lodging susceptibility	**	0.639	NS	0.980
Individual seed weight	**	0.879	**	0.953

^a^ NS and **: not significant at *P* < 0.05 and significant at *P* < 0.01, respectively; *r*_*g*_ always different from zero (*P* < 0.01)

**Fig. 1:**
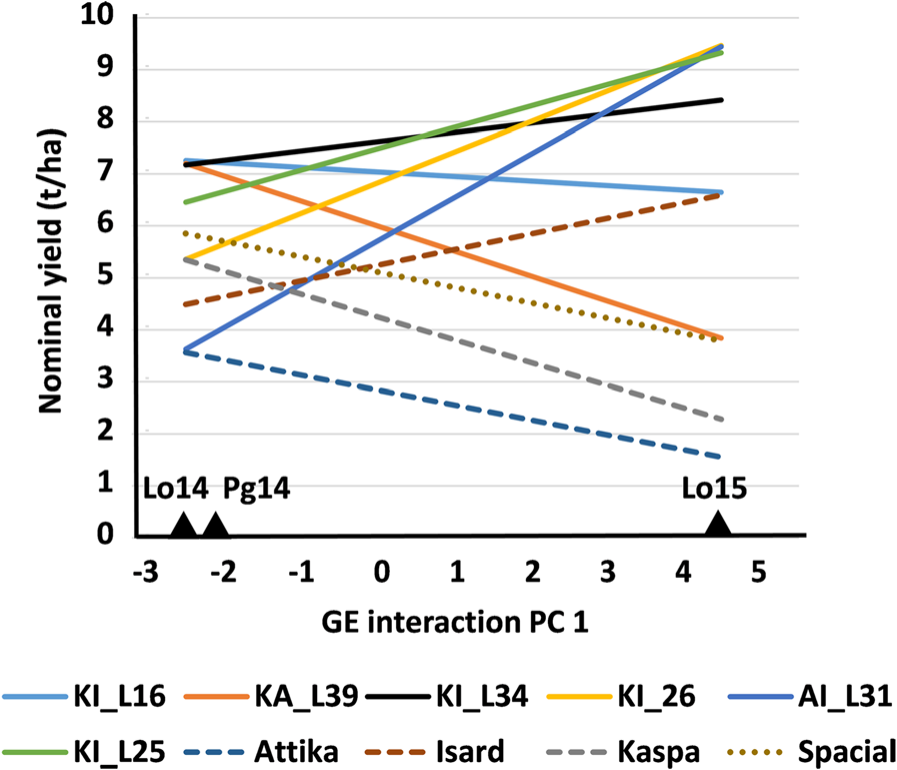
Nominal grain yield of six top-performing pea inbred lines out of 306 derived from three connected crosses, three parent cultivars (Attika, Isard, Kaspa) and one commercial cultivar (Spacial) as a function of the environment score on the first genotype × environment interaction principal component axis (PC 1) [environments are Lodi 2013–14 (Lo14), Lodi 2014–15 (Lo15), and Perugia 2013–14 (Pg14); the graph includes the two top-yielding lines in each environment or across environments]

The first GEI PC axis was the only significant one at *P* < 0.05 and accounted for 87% of the GEI variation in the AMMI analysis for grain yield. The environment ordination along this axis highlighted the contrast between the cold-prone environment and the other environments for genotype responses (Fig. 1). The AMMI-modelled yield responses revealed marked GEI of cross-over type (i.e., implying rank change) between top-yielding lines across contrasting environments, along with wider line variation in the cold-prone environment (Fig. 1). Various RILs exhibited a clear yield advantage not only over their parent lines but also over the control variety Spacial (Fig. 1).

Higher grain yield in the cold-prone environment was strictly associated (*P* < 0.001) with greater winter plant survival not only for lines of the population K × A (*r* = 0.78) but also for those of the other RIL populations (*r* ≥ 0.70). In the population K × A, later onset of flowering was associated with greater winter survival (*r* = 0.28, *P* < 0.01), as well as higher grain yield in any environment (*r* ≥ 0.41; *P* < 0.01). Correlations for other traits assessed for each RIL in each environment were modest and largely inconsistent.

### Assessment of genomic selection models and data configurations

Next generation sequencing produced on average 551,210 reads per sample. The dDocent pipeline produced a mock reference genome of 74,831 contigs, with an average length of 94.46 bases. The actual number of polymorphic SNP markers was heavily affected by allowed missing rate, which ranged from 6058 for missing rate of 1% to 18,057 for missing rate of 30%) (Additional file 3: Figure S1).

**Fig. 2:**
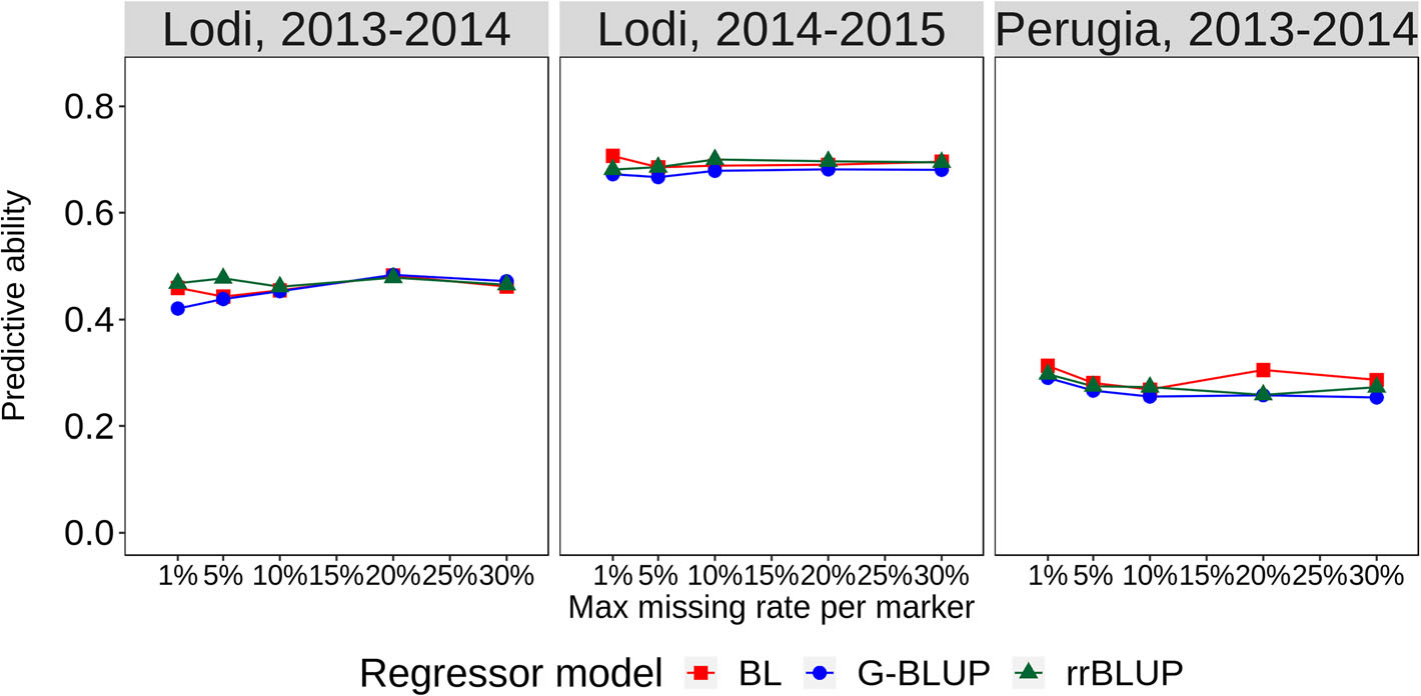
Intra-population predictive ability for pea grain yield in three environments, for all combinations of three regression models (BL, Bayesian Lasso; rrBLUP, Ridge regression BLUP; G-BLUP, genomic BLUP) and five genotype missing data thresholds. Data averaged across three pea RIL populations and 50 repetitions of 10-fold stratified cross-validation per individual analysis

**Fig. 3:**
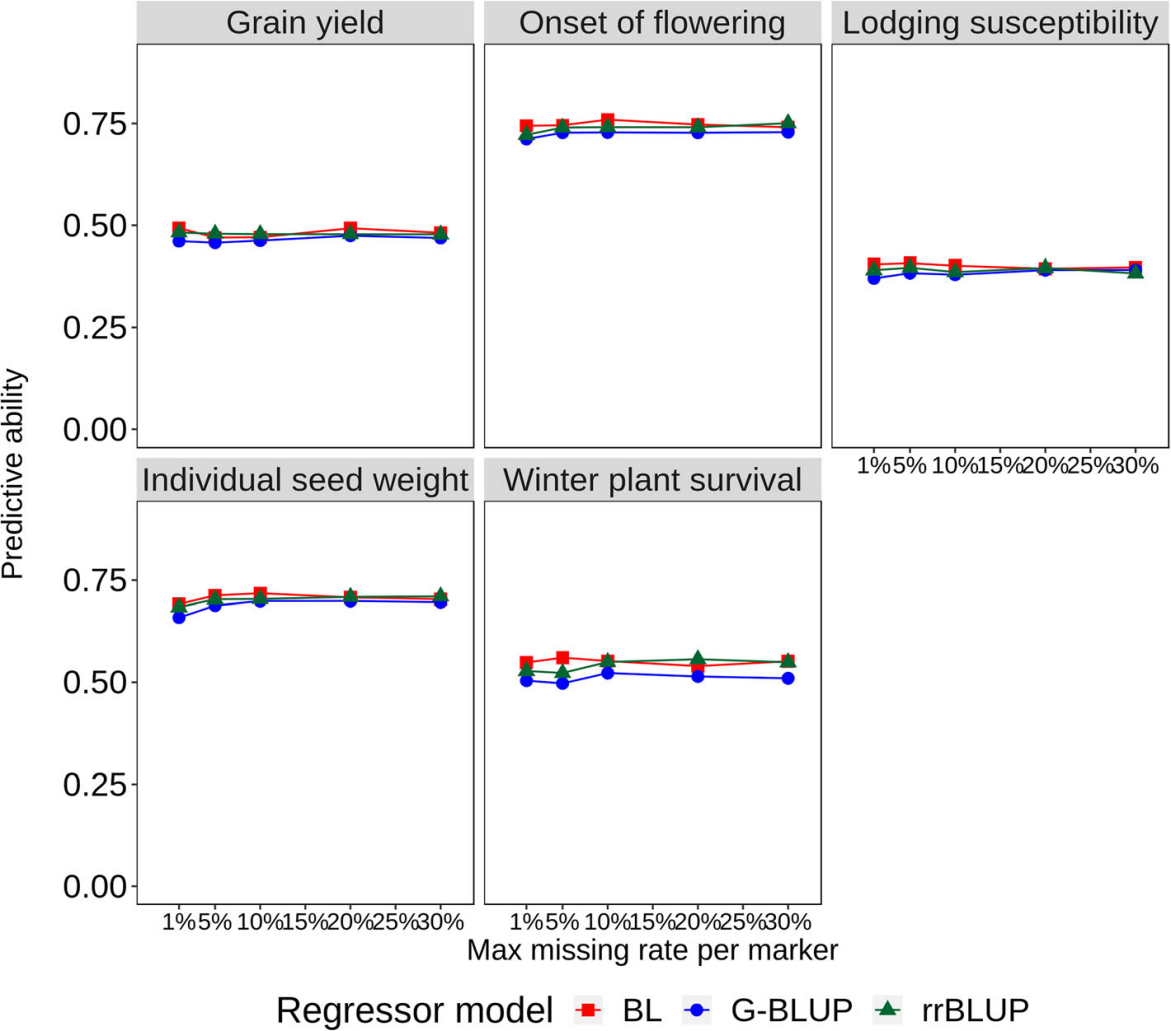
Intra-population predictive ability for pea mean grain yield, onset of flowering, lodging susceptibility and individual seed weight across three environments and winter plant survival in one environment, for all combinations of three regression models (BL, Bayesian Lasso; rrBLUP, Ridge regression BLUP; G-BLUP, genomic BLUP) and five genotype missing data thresholds. Data averaged across two (lodging susceptibility) or three (other traits) RIL populations and 50 repetitions of 10-fold stratified cross-validation per individual analysis

The value averaged across RIL populations of intra-population, intra-environment predictive ability for grain yield in each environment based on cross-validations is reported in Fig. 2 for different models and data configurations. In general, the three GS models performed very similarly, and exhibited just a small increase of predictive ability as a function of missing data threshold. Predictions for yield were high for the cold-prone environment (Lodi 2014–15) (with correlation between GS-modelled and observed data for the best model and data configuration *r* = 0.707), moderately high for Lodi 2013–14 (maximum *r* = 0.484), and fairly modest for Perugia 2013–14 (maximum *r* = 0.313). Moderately high predictive ability was obtained as well for line mean yield across environments (maximum *r* = 0.493, using Bayesian Lasso with missing rate = 1%; Fig. 3).

The values averaged across RIL populations of intra-population, intra-environment predictive ability for mean onset of flowering, lodging susceptibility and seed weight across three environments and winter plant survival in one environment are reported in Fig. 3 for different GS models and data configurations. A slight advantage of Bayesian Lasso over the other models emerged for onset of flowering and winter plant survival. A plateau of predictive ability was achieved between 1 and 10% missing data for all traits. Correlations between GS-modelled and observed data for best GS models indicated that onset of flowering and seed weight were highly predictable (*r* > 0.70), whereas grain yield, lodging susceptibility and winter plant survival exhibited moderate predictive ability (*r* > 0.40).

GS model choice for following analyses was based on Root Mean Square Difference (RMSD) values between the predictive ability of the best trait-specific GS model and each available combination of models and missing data thresholds, averaging the results across RIL populations and traits (Additional file 4: Tables S3 and S3bis). Bayesian Lasso with missing data threshold = 1% (including 6058 polymorphic SNP markers) was top-ranking according to this criterion, displaying in all cases just a modest penalty in terms of *r* value (< 0.04) in comparison with the best-predicting GS model and data configuration for the specific trait-environment combination. This assessment indicated the following ranking of GS models for predictive ability, albeit with small differences in average RMSD: Bayesian Lasso > rrBLUP > G-BLUP (Additional file 4: Table S3bis).

Model training on all RIL populations (without structure information) rather than on individual populations allowed for greater predictive ability for all traits, with the exception of winter plant survival (Table 5). The addition of structure information led to nil or negligible increase of predictive ability, with the exception of winter plant survival (Table 5). GS training on all RIL populations without structure imputation was preferred for all subsequent analyses, because of its similar predictive ability and wider applicability to breeding material in comparison with the model with structure information.

**Table 5:** Intra-population predictive ability (PA) for pea grain yield, onset of flowering, lodging susceptibility and individual seed weight averaged across three environments and winter plant survival in one environment, for different Bayesian Lasso model training and account of population structure. Values averaged across two (lodging susceptibility) or three (other traits) RIL populations

Trait	Training^a^	Structure^b^	PA
Grain yield	Single	No	0.452
	All	No	0.476
	All	Yes	0.474
Onset of flowering	Single	No	0.710
	All	No	0.747
	All	Yes	0.749
Lodging susceptibility	Single	No	0.385
	All	No	0.404
	All	Yes	0.411
Individual seed weight	Single	No	0.695
	All	No	0.696
	All	Yes	0.700
Winter plant survival	Single	No	0.561
	All	No	0.557
	All	Yes	0.585

^a^ Single = model trained on the specific population; all = model trained on all populations joined in a single data set. Fifty repetitions of 10-fold stratified cross-validation per individual analysis

^b^ No = no structure information; yes = structure information as RIL population fixed factor

### Assessment of inter-environment and inter-population genomic predictions

Intra-population, inter-environment predictive ability values averaged across the three RIL populations are reported in Table 6 for all traits and environments. The three environments did not differ largely for predictive ability when respectively used as the only training environment. The only exception was represented by poorer predictions for lodging susceptibility issued by Lodi 2014–15 compared with the other environments, which was associated with distinctly narrower trait variation (Table 2) and more widespread lodging (Table 1). Onset of flowering and seed weight, which featured high intra-environment predictive ability and low GEI, exhibited high inter-environment predictive ability (average *r* > 0.63; Table 6). Grain yield and lodging susceptibility displayed relatively low average inter-environment predictive ability (0.28 ≤ *r* ≤ 0.30, Table 6).

**Table 6:** Inter-environment predictive ability for four pea traits, using Bayesian Lasso modelling trained in one environments for prediction of independent lines in each of two other environments. Values averaged across two (lodging susceptibility) or three (other traits) RIL populations

	Training environment^b^			
Trait^a^	Lodi 2013–14	Lodi 2014–15	Perugia 2013–14	Average
GY	0.311	0.240	0.336	0.296
OF	0.680	0.614	0.669	0.654
LS	0.386	0.180	0.269	0.278
SW	0.668	0.616	0.629	0.638

^a^ GY, grain yield; OF, onset of flowering; LS, lodging susceptibility; SW, individual seed weight

^b^ Fifty repetitions of 10-fold stratified cross-validation per individual analysis

On average, the reduction of inter-environment predictive ability due to the use of different populations for GS model training and model application (as assessed by comparing inter-population, inter-environment prediction in Table 7 vs intra-population, inter-environment predictions in Table 6) was substantial for all traits. It amounted to 37% (0.187 vs 0.296) for grain yield, 39% for onset of flowering, 56% for seed weight, and 20% for lodging susceptibility. In general, inter-population predictions for grain yield and onset of flowering tended to be lower when using the population derived from the two European parent cultivars (A × I) as the training set (Table 7). Inter-population predictions for winter plant survival, which could only be assessed for the intra-environment scenario, were very low for the model trained on the population K × A that excluded the cold-tolerant parent genotype Isard (Table 7).

**Table 7:** Inter-population predictive ability for same (intra-environment) or other environments (inter-environment) for five pea traits, using Bayesian Lasso modelling trained in one RIL population for predictions in one (lodging susceptibility) or two (other traits) other RIL populations, averaging results across validation RIL populations in one (winter plant survival) or three (other traits) environments. Inter-environment predictions for models trained in one environment and tested in each of two other environments, averaging results across training environments

	Intra-environment^b^				Inter-environment^b^			
Trait^a^	A × I	K × A	K × I	Average	A × I	K × A	K × I	Average
GY	0.092	0.282	0.367	0.247	0.042	0.226	0.292	0.187
OF	0.359	0.455	0.522	0.445	0.287	0.454	0.457	0.399
LS	0.296	–	0.333	0.315	0.195	–	0.250	0.223
SW	0.303	0.253	0.322	0.293	0.274	0.263	0.296	0.277
WS	0.230	0.130	0.410	0.257	–	–	–	–

^a^ GY, grain yield; OF, onset of flowering; LS, lodging susceptibility; SW, individual seed weight; WS, winter plant survival

^b^Column headings indicate the training population

The average reduction of inter-population predictive ability passing from intra-environment to inter-environment predictions was substantial for grain yield (24%; 0.187 vs 0.247) and lodging susceptibility (20%), and modest (≤ 10%) for the two traits featuring small GEI, i.e., onset of flowering and seed weight (Table 7).

### Comparison of genomic vs phenotypic selection

On average, the GS modelling of line responses for grain yield and lodging susceptibility based on phenotyping data in two environments exhibited somewhat higher correlation with phenotypic data in an independent environment than the phenotypic data themselves used for GS modelling (Table 8). Phenotypic and GS-modelled data displayed nearly identical correlation with independent phenotypic data in the case of flowering time and seed weight (Table 8).

**Table 8:** Correlation (*r*) of phenotypic data or genomic selection (GS)-modelled data based on two (test) environments with phenotypic data in another (validation) environment, square root of broad-sense heritability (*H*) when adopting two test environments, accuracy (*r*_*Ac*_) of GS modelling trained in two environments for prediction in one validation environment, and GS vs phenotypic selection (PS) efficiency ratio based on predicted genetic gains per unit time for similar evaluation costs assuming two environments for PS and for generation of phenotyping data for intra-population (GS_A_) and inter-population (GS_B_) GS scenarios, for four pea traits. Data averaged across three environment combinations and two (lodging susceptibility) or three (other traits) RIL populations

	*r*				GS_A_/PS efficiency ratio^e^			GS_B_/PS efficiency ratio^e^	
Trait^a^	Phenotypic data	GS-modelled data^b^	*H* ^c^	GS_A_ *r*_*Ac*_^d^	*t*_*P*_ = 1	*t*_*P*_ = 2	GS_B_ *r*_*Ac*_^f^	*t*_*P*_ = 1	*t*_*P*_ = 2
GY	0.377	0.402	0.632	0.390	1.801	3.602	0.262	1.209	2.418
OF	0.837	0.827	0.929	0.690	2.170	4.340	0.445	1.398	2.796
LS	0.398	0.436	0.609	0.485	2.323	4.647	0.420	2.014	4.029
SW	0.831	0.836	0.932	0.723	2.266	4.531	0.327	1.024	2.049

^a^ GY, grain yield; OF, onset of flowering; LS, lodging susceptibility; SW, individual seed weight

^b^ Using Bayesian Lasso modelling trained on all genotype data

^c^ Assuming experiments with three replicates (as the current phenotyping experiments)

^d^ Using Bayesian Lasso modelling for prediction of independent lines, using 50 repetitions of 10-fold stratified cross-validation per individual analysis

^e^ As ratio (*i*_*G*_ *r*_*Ac*_ / *t*_*G*_) / (*i*_*P*_ *H* / *t*_*P*_), where *i*_*G*_ and *i*_*P*_ are standardized selection differentials for GS and PS, respectively, setting *i*_*G*_ = 1.46 *i*_*P*_ to approach same evaluation costs; and *t*_*G*_ and *t*_*P*_ are cycle duration for GS and PS, setting *t*_*G*_ = 0.5 and *t*_*P*_ = 1 (two test sites in the same test year) or *t*_*P*_ = 2 (two test years in the same site)

^f^ Using Bayesian Lasso model training on data of one RIL population for prediction within each of two other populations

Compared with onset of flowering and seed weight, grain yield and lodging susceptibility exhibited lower intra-population predictive accuracy (for an independent environment) of GS-modelled data constructed from phenotyping data of two environments, as well as lower broad-sense heritability for PS based on two environments (Table 8). As a result, the comparison of GS vs PS based on expected gains produced fairly similar results for the four traits (Table 8). GS was predicted to be at least 80% more efficient than PS when the latter contemplated two selection environments from one test year, and at least 3.6 fold more efficient when PS was based on two environments from different years (Table 8).

The average reduction of predictive accuracy arising from inter-population instead of intra-population application of GS predictions amounted to 33% for grain yield (0.262 vs 0.390), 36% for onset of flowering, 13% for lodging susceptibility, and 55% for seed weight (Table 8). However, the inter-population GS scenario displayed higher selection efficiency than one year-based PS for all traits (although marginally for seed weight), while showing at least two-fold greater efficiency for all traits when PS spanned over two years (Table 8). It is noteworthy that even in the worst scenario for GS (inter-population GS predictions vs one-year PS), GS displayed 20% greater predicted efficiency than PS for yield selection.

### Genome-wide association study

**Fig. 4:**
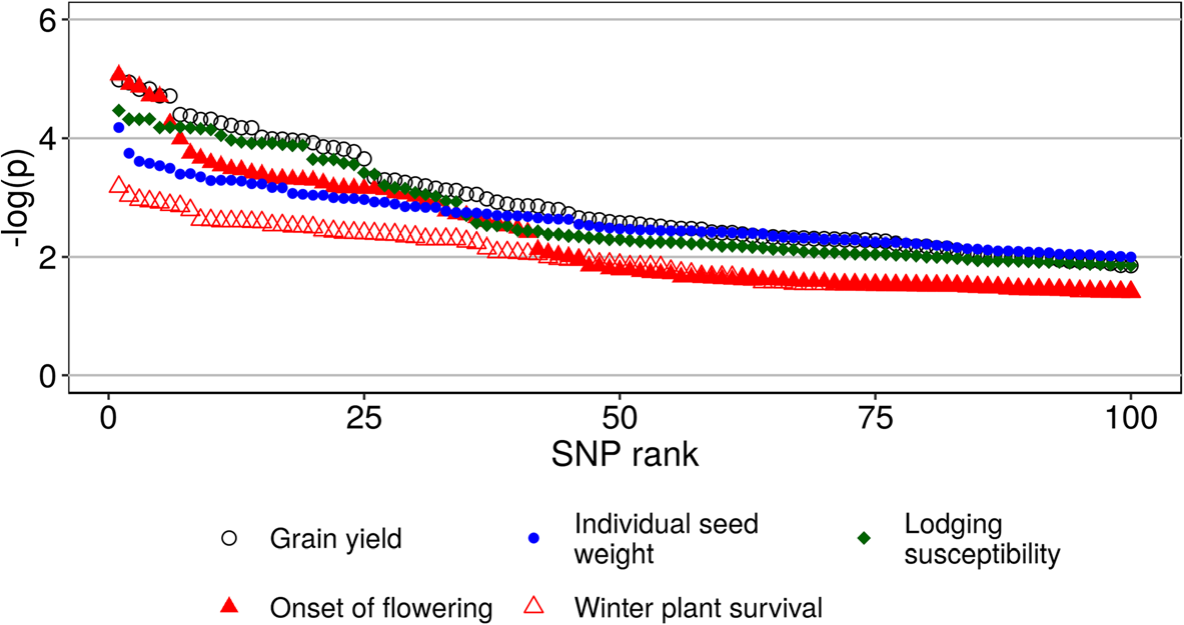
Top 100 genome-wide association scores, ranked in descending order, for single-nucleotide polymorphism (SNP) markers associated with five phenotypic traits of pea. GWAS was performed on stratified data, with each of two (lodging susceptibility) or three (other traits) RIL populations acting as a stratum

The GWAS performed on stratified data of the three RIL populations identified, at the association score threshold of three, 30 GBS-generated markers associated with onset of flowering, 36 with grain yield, 32 with lodging susceptibility, 21 with seed weight, and two with winter survival. The association scores are graphically summarized in Fig. 4.

Additional file 5: Table S4 reports detailed information on marker ranking and association scores for each significant marker-trait association, as well as results of the linkage analysis of these markers with the Illumina array markers used for a consensus map in [42]. The absence of high marker-trait linkage in the presence of genetic variation confirmed indirectly the highly polygenic control of grain yield, lodging susceptibility and seed weight. The opposite trend was manifest for flowering time, where most highly-linked SNP markers concentrated in a genomic area of LG II, based on the high (*r*^*2*^ > 0.8) or medium (*r*^*2*^ > 0.4) linkage of 9 markers with the arabinase A encoding gene *ARBA1788,* 8 with the cell wall invertase 1 gene *CWi1*, and 10 with the Glutathione S-transferase gene F3586 (Additional file 5: Table S4). Out of the 36 SNP markers highly associated with grain yield, 24 were also associated with onset of flowering, exhibiting a genetic association that agreed with the moderate phenotypic correlation between the two traits across RIL populations (*r* = 0.38, *P* < 0.001). Out of the 32 SNP markers highly associated with lodging susceptibility, 14 were placed in a genomic region of LG V, based on their high linkage with the beta-1,3 glucanase encoding gene *gns2* and the ABA insensitive 3 gene *Abi3*. The markers highly associated with seed weight were placed in two genomic areas of LG VII, one at the beginning of LG VII based on associations with the Sulfate transporter gene *St100044*, and the other located around the middle of LG VII based on associations with the Acetohydroxy acid isomeroreductase gene *Acetisom*. No genes were linked with the two markers highly associated with winter survival.

## Discussion

### Phenotypic variation, genotype × environment interaction and trait interrelationships

The main limitation of this study lies in the limited number of test environments it was based upon. However, the observed GEI pattern for pea grain yield, implying modest GEI across geographically-distant Italian locations and high GEI across test years as a consequence of marked year-to-year variation for low winter temperatures, confirmed earlier results relative to different sets of recent varieties grown in a larger number of Italian environments [34, 36]. Those studies and the current one indicate the possible occurrence in northern Italy of absolute minimum temperatures in winter below the threshold of − 8.5 °C that was reportedly associated with sizeable plant mortality of relatively winter-hardy pea [43]. The year-to-year variation for this and other climatic variables is expected to increase as consequence of climate change [44]. The reported GEI pattern supports the breeding for wide adaptation to Italian environments, given the limited scope for exploiting specific adaptation to distinct geographic areas [45]. The GEI pattern has implications as well for genomic selection modelling. GS models that incorporates GEI effects have been developed [46], but their application in the current GEI scenario has limited practical interest, because the exploitation of genome-enabled predictions for a specific year (e.g., a cold-prone one) is prevented by the impossibility to know in advance how a future year will be like. The reported ability of single environments to predict (genomically or phenotypically) the genotype responses in other target environments is the information of practical interest for the current wide-adaptation prospect.

The cold-prone environment had a greater impact on the evaluation of line breeding values across environments than the other environments (as shown by the amplification of line yield variation according to CV_g_ values in Table 2 and AMMI analysis results in Fig. 1), supporting the adoption of multi-year PS for yield to increase the probability to encounter substantial cold stress. However, multi-year PS implies delayed variety selection, reinforcing the potential interest of genome-enabled models aimed to select more timely for winter plant survival and wide adaptation. The interest of GS to shorten the field-based evaluation would be less pronounced for onset of flowering, seed weight and susceptibility to lodging, which were less affected by GEI and more heritable than grain yield according to current and earlier findings [34].

The current RIL populations, which derived from crosses between elite varieties, represented well the type of material generated by breeding programs. Indeed, some lines displayed distinct yield progress not only over their parent lines but also over the elite commercial standard represented by the variety Spacial. The wider within-population than between-population variation observed in these elite RIL populations encourages the perspective application of GS to lines of a reduced number of RIL populations. However, within-population variation was smaller than between-population variation with respect to GEI effects for yield (Table 3). This result paralleled the smaller within-population than between-population variation for cold tolerance (Table 3), i.e., the trait that had a major impact on line specific adaptation to the cold-prone environment and, thereby, on GEI effects. These latter findings suggest that multi-environment evaluation of RIL populations as bulked progenies could provide useful indications on the adaptation pattern of most of their component lines.

The modest or nil correlation of winter plant survival with onset of flowering emerged already for other autumn-sown pea material grown in Italian environments [15]. In agreement with earlier findings [34], most winter-type material combined cold tolerance with relatively early onset of flowering, because its main cold tolerance mechanism is not cold stress escape by a late phenology but the display of a rosette-like winter growth habit that features cold-tolerant germplasm in pea and other grain legume species [14, 15] and can contribute to cold tolerance by its relationships with lower relative plant water content [47]. Grain yield and onset of flowering exhibited a moderate positive correlation in the current environments, in contrast with the negative correlation that emerged for the same RIL populations grown under severe terminal drought in a phenotyping platform [13] or under spring sowing in northern Italy [42] (where early flowering was important to escape terminal drought and heat stress in the absence of winter cold stress).

### Genomic selection

The similar performance of the tested GS models, and the negligible or nil increase of predictive ability arising from imputing population structure information, agreed with earlier results for pea [13, 30]. The higher intra-population, intra-environment predictive ability reported for grain yield of the same RIL material under severe terminal drought [13] relative to the current environments (0.72 vs 0.48 for best predictions averaged across populations) could be attributed to the fact that line yield responses in that study were ecologically simpler, owing to their close relationship with drought stress escape via early onset of flowering. The higher predictive accuracy for pea yield reported for Italian environments in a preliminary study [32] was less realistic than the current one, because phenotyping in two test sites of the same year excluded the complication of genotype × year interactions from predictions. The dDocent pipeline provided a larger number of SNP markers than the UNEAK pipeline used in our earlier studies [13, 32].

The assessment of inter-environment predictive abilities for models trained on one-environment data provided indications on the variation between environments for ability to generate useful phenotypic information for GS modelling. Onset of flowering and seed weight stood forward as well-predictable traits in agreement with earlier findings [30, 31], owing to low exposure to GEI and intrinsically high predictive ability (as highlighted by intra-population prediction of line mean responses across environments in Fig. 3). Grain yield exhibited predictive ability values in the range of those reported for another inbred species such as wheat [40], which, although fairly low, proved of interest for GS when compared with PS opportunities. For all of these traits, the similar predictive ability displayed by the single training environments (Table 6) was comforting, suggesting that every environment may produce useful phenotyping data for GS modelling. The prediction of lodging susceptibility proved more difficult, especially because of poor predictions arising from model training on Lodi 2014–15 data (Table 6). The widespread lodging occurred in this cold-prone environment, which leveled out the phenotypic variation, was probably a consequence of less over-wintering plants, since a dense canopy favors plants’ standing ability via greater intertwining of tendrils.

We envisaged the exploitation of inter-population predictions only for RIL populations that share one parent with the RIL population used for model training - which implies greater potential success relative to the case of no common parent. As expected, inter-environment predictions were substantially worse than intra-population predictions. The trend towards poorer inter-population predictions issued by the training population A × I relative to the other RIL populations (Table 7) was probably due to the genetic similarity of its European parent cultivars and their difficulty to properly account for the genetic contribution of the more exotic and dissimilar Australian cultivar. Indeed, Nei’s [48] distance based on the SNP markers used for inter-population predictions was 0.09 between Attika and Isard, while being ≥ 0.14 between each of these cultivars and Kaspa. Hence, RIL training populations derived from genetically-distant parents may prove more useful for inter-population predictions.

GS selection for winter plant survival could not undergo a thorough assessment, owing to meaningful trait observation in only one environment. The moderately high intra-population, intra-environment predictive ability displayed by GS for this trait (Fig. 3) was encouraging, but inter-population predictions may be inaccurate according to our results (Table 7).

The slightly higher correlation with phenotypic data in an independent environment exhibited by GS-modelled data of grain yield or lodging susceptibility compared to the phenotypic data they were based upon (Table 8) is remarkable, suggesting the ability of the GS modelling process to reduce the noise that is present in phenotyping data. Better correlations of GS-predicted yield data than phenotypic data emerged already for early-generation evaluation of wheat lines across different test years [49]. GS-modelled and phenotyping data displayed similar correlation with data in an independent environment for onset of flowering and seed weight, in agreement with earlier results for pea [31].

The average intra-population prediction accuracy of GS models trained in two environments was always lower than the broad-sense heritability relative to PS based on two selection environments (Table 8). However, the advantages provided by GBS-based GS over PS in terms of shorter selection cycle and more evaluated lines for same cost led to distinctly greater predicted efficiency of GS over PS for nearly any trait and selection scenario (Table 8). When considering two years for grain yield and one year for the other traits as the likely duration cycle for PS (as suggested by GEI results), GS compared with PS would be (i) 3.6-fold more efficient for yield and over 2-fold more efficient for the other traits, when using intra-population predictions, and (ii) 2.4-fold more efficient for yield and more efficient for the other traits, when using inter-population predictions. This assessment was influenced by our estimate of five-fold higher cost of PS relative to GS [28], which coincided, anyway, with estimates for wheat breeding based on the same number of total plots per field-evaluated line [50]. The same wheat study estimated three- to five-fold greater expected efficiency of GS over PS for grain yield selection, values that are more favourable for GS than the current ones.

### Genome-wide association study

The main objective of the GWAS study was to shed some light onto the genetic control of the target traits. Highly polygenic traits, whose control depends on many quantitative trait loci (QTL) featuring small genetic effects, are expected to display loose marker-trait associations, as it was the case for grain yield, lodging susceptibility and seed weight. The improvement of such traits is expected to be more efficient via genomic selection than via marker-assisted selection.

The GWAS confirmed at the genetic level the moderate positive relationship between onset of flowering and grain yield that was observed phenotypically. Many of the markers associated with both traits were related to a genomic region in LG II that was reported as a major QTL for flowering time in earlier studies [42, 51]. The co-location of this QTL with grain yield emerged already in two earlier studies [13, 42] in which, however, the relationship of grain yield with onset of flowering was negative rather than positive, according to the adaptive value of early flowering in environments featuring severe terminal drought and heat stress and lack of winter cold stress.

## Conclusions

This study supports the implementation of GS as a means to increase, for same costs, the efficiency of pea breeding for wide adaptation to Italian environments. Along with earlier studies for pea [13, 32], soybean [52, 53], alfalfa [41, 54] and chick pea [55], it provides evidence for the value of GS for legume crop yield improvement. Interestingly, GS was found more efficient than PS even for traits that are genetically simpler, more heritable and less subjected to GEI than grain yield, such as seed weight and onset of flowering, because they proved as well more predictable genomically. The wider within-population than between-population variation observed for elite RIL populations justifies the perspective application of GS to a large number of lines belonging to a fairly small number of RIL populations derived from elite parent genotypes, to increase the probability to pick up the extremely rare genotypes featuring the desired recombination of several favourable alleles for each of various traits. Our envisaged use of GS implied the development of a specific GS model for each elite RIL population that would become progressively available from crosses between new elite parents (where elite populations could be selected out of several populations preliminary evaluated phenotypically as bulked material). To limit the GS model training effort, we also envisaged the application of available GS models to new RIL populations having one parent in common with the RIL used for model construction. Our results showed that inter-population predictions for this context, albeit not as well-performing as intra-population predictions, could efficiently contribute to grain yield selection.

## Methods

### Plant material

Our study included 306 genotypes belonging to three connected RIL populations originated from paired crosses between Attika (a European cultivar described as a spring-type), Isard (a French winter-type cultivar) and Kaspa (an Australian cultivar of Mediterranean type). The three parent cultivars, each belonging to a different phenological type, were chosen in the crossing program because they displayed high and stable grain yield and only moderate phenological differences across environments of northern and southern Italy [34, 56]. The RIL populations are coded henceforth as A × I, K × A and K × I from the initials of their respective parents. The population A × I included 102 lines; the population K × A, 100 lines; and the population K × I, 104 lines. Four F_6_ plants per line were grown in a non-heated glasshouse to collect DNA samples for line genotyping and to produce seed, which underwent one additional generation of multiplication before use for phenotyping. In addition, the evaluation work included the three parent cultivars, as well as the cultivar Spacial that displayed excellent adaptation across organically-managed environments of northern and central Italy in recent variety trials [36].

### Phenotyping

The 306 genotypes were evaluated under rain-fed conditions in three autumn-sown environments of northern or central Italy, named hereafter as Lodi 2013–14, Lodi 2014–15 and Perugia 2013–14 after their site name and cropping year (referred as ‘year’ hereafter). The site of Lodi (45°19′N, 9°30′E), representative of the subcontinental climate typical of northern Italy, included two environments, to sample the wide year-to-year climatic variation for extent of winter low temperatures of this area (Additional file 1: Table S1). Lodi’s environments represented the two major crop managements for pea in Italy, namely, organic (2013–14) and conventional cropping (2014–15). The environment of Perugia (43°06′N, 12°23′E), which was characterized by a cool Mediterranean climate that is widespread in central Italy and inland southern Italy (Additional file 1: Table S1), was organically-managed, to ensure that possible GEI effects across the two sites could be attributed to the different climatic region with no bias arising from different crop management or cropping year.

Each experiment was laid out in a randomized complete block design with three replications. The three parent lines and Spacial were replicated thrice within each block. Each plot had 0.96 m^2^ size and included four rows 1.2 m long, 0.2 m apart. Fifteen seeds per row (overall seed density = 62.5 seeds/m^2^) were sown at 3 cm depth by a pneumatic seed drill. Pre-sowing mineral fertilization (24 kg/ha N, 72 kg/ha P_2_O_5_, and 72 kg/ha K_2_O), and chemical weed control [Stomp® 330 E (a.i. Pendimethalin at 307 g/L) at 4.5 L/ha], were applied only in the conventionally-managed environment (Lodi 2014–15). Seedbed preparation with ploughing and harrowing was the same in conventional and organic conditions. Sowing took place on November 7 for Lodi 2013–14, October 22 for Lodi 2014–15, and November 25 for Perugia 2013–14. No mechanical weed control was applied under organic management. The crop was always harvested by combine within the first ten days of June.

The following traits were recorded on a plot basis: (i) winter plant survival, based on plant counts at the onset and at the end of winter; (ii) lodging susceptibility, visually assessed at maturity on a 5-level scale ranging from 1 = lodging limited to the basal part of the stem to 5 = complete lodging; (iii) onset of flowering, as the number of days after April 1 when 50% of plants in the plot had at least one fully open flower; (iv) dry grain yield, after combine-harvesting the plot and assessing seed moisture on a random sample of 250 seeds oven-dried at 90 °C for four days; (v) individual dry seed weight, assessed on the seed sample used for seed moisture determination.

### Statistical analysis of phenotypic data

The following analyses were performed for each trait in each environment. An analysis of variance (ANOVA) assessed the variation among genotypes within each RIL population. Components of variance relative to variation among genotypes (*S*_*G*_^*2*^) and experimental error (*S*_*e*_^*2*^) were estimated for each RIL population by a restricted maximum likelihood (REML) method, expressing the within-population genetic variation in terms of genetic coefficient of variation [CV_g_ = (*S*_*G*_/*m*) × 100, where *m* = trait mean value]. Components of variance estimated for the pooled genotypes of the three populations were used to compute best linear unbiased prediction (BLUP) values for each trait according to DeLacy et al. [57], which were used as phenotyping data for subsequent GS analyses. The following ANOVA including the fixed factor RIL population and the random factors genotype within RIL population and block aimed to compare the RIL populations for trait mean values in each environment:

$$ {Y}_{kir}=m+{R}_k+{G}_i\ \left({R}_k\right)+{B}_r+{e}_{kir} $$

where *Y*_*kir*_ is the observed response of the genotype *i* belonging to the RIL population *k* in the block *r*; *m* is the grand mean; *R*_*k*_, *G*_*i*_ and *B*_*r*_ are RIL population, genotype and block main effects, respectively; and *e*_*kir*_ is the random error.

Other analyses were carried out for line values of the RIL populations across environments. Components of variance were estimated by two separate analyses for the following sources of variation held as random factors, using a REML method: (i) pooled genotypes of the RIL populations (*S*_*G*_^*2*^), GEI (*S*_*GE*_^*2*^), and experimental error (*S*_*e*_^*2*^); (ii) RIL population (*S*_*R*_^*2*^), genotype within RIL population (*S*_*G(R)*_^*2*^), RIL population × environment interaction (*S*_*RE*_^*2*^), genotype within RIL population × environment interaction (*S*_*G(R)E*_^*2*^), and experimental error (*S*_*e*_^*2*^). The aim of the latter analysis was to estimate the relative extent of variation between and within populations. ANOVAs including the same factors (along with environment and block) were used to test the difference from zero of the variance components. For example, the ANOVA model for the latter analysis was:

$$ {Y}_{kijr}=m+{R}_k+{G}_i\ \left({R}_k\right)+{E}_j+{B}_r\ \left({E}_j\right)+{R}_k\ {E}_j+{G}_i\ \left({R}_k\right)\ {E}_j+{e}_{kijr} $$

where *Y*_*kijr*_ is the observed response of the genotype *i* belonging to the RIL population *k* in the block *r* of the environment *j*; *m*, *R*_*k*_, *Gi* and *B*_*r*_ correspond to prior notations; *R*_*k*_ *E*_*j*_ and *G*_*i*_ *(R*_*k*_*) E*_*j*_ are RIL population × environment and genotype within RIL population × environment interaction effects, respectively; and *e*_*kijr*_ is the random error.

Further ANOVAs including the random factors genotype and block and the fixed factor environment aimed to assess the occurrence of GEI for the pooled genotypes across subsets of environments represented by (i) years (2013–14 and 2014–15) in the same location (Lodi), and (ii) locations (Lodi and Perugia) in the same year (2013–14). The consistency of genotype responses across these pairs of environments was assessed in terms of genetic correlation *r*_*g*_ as [58]:

$$ {r}_g=r/\left({H}_1\ {H}_2\right) $$

where *r* is the phenotypic correlation for genotype value across the relevant pair of environments, and *H*_*1*_ and *H*_*2*_ are the square root of the broad-sense heritability on a genotype mean basis (*H*^*2*^) for the two environments. Each *H*^*2*^ value was computed from variance components for genotype (*S*_*G*_^*2*^) and experimental error (*S*_*e*_^*2*^) of the specific environment estimated by a REML method, and the number of experiment replications *n*, as:

$$ {H}^2={S_G}^2/\left({S_G}^2+{S_e}^2/n\right). $$

For each trait and RIL population, we estimated the broad-sense heritability on a genotype mean basis across the three environments from components of variance relative to genotype (*S*_*G*_^*2*^), GEI (*S*_*GE*_^*2*^) and experimental error (*S*_*e*_^*2*^) as:

$$ {H}^2={S_G}^2/\left({S_G}^2+{S_{GE}}^2/e+{S_e}^2/e\ n\right) $$

where *e* is the number of environments.

Grain yield data from all genotypes, including the four reference cultivars, underwent a combined ANOVA in which the GEI variation was partitioned by Additive Main effects and Multiplicative Interaction (AMMI) analysis [59]. This analysis, which has special value for revealing GEI patterns [60], aimed to highlight the extent of GEI among top-yielding lines and the performance of these lines relative to their parent genotypes and the elite commercial cultivar Spacial, as well as providing additional information on the similarity of test environments for GEI effects. AMMI analysis describes the response *Y*_*ij*_ of the genotype *i* in the environment *j* (omitting the block factor) according to the following model [59]:

$$ {Y}_{ij}=m+{G}_i+{L}_j+\sum \left({u}_{in}\surd {l}_n\right)\ \left({v}_{jn}\surd {l}_n\right)+{d}_{ij} $$

where *m*, *G*_*i*_ and *L*_*j*_ correspond to earlier notations, whereas *GL*_*ij*_ interaction effects are partitioned into (i) the pattern component, in which *u*_*in*_ and *v*_*jn*_ are eigenvectors (scaled as unit vectors, i.e. ∑ *u*_*i*_^2^ = ∑ *v*_*j*_^2^ = 1) of the genotype *i* and the location *j*, respectively, and *l*_*n*_ is the singular value, for the principal component (PC) axis *n*, and (ii) the noise component *d*_*ij*_, indicating the deviation from the model. Data from each reference cultivar were averaged across the three replicates within each block prior to data analysis. The number of GEI principal component (PC) axes included in the AMMI model was defined as recommended in [61], namely, by testing for statistical significance the *d*_*ij*_ term by the *F*_*R*_ test [61] starting from the model with *n* = 0, and adding one PC axis whenever *d*_*ij*_ was statistically (*P* < 0.05) different from zero. AMMI-modelled yield responses were graphically displayed as a function of the environment PC 1 score as nominal yields, which sum up the estimated entry mean value and the modelled GEI effect on PC 1 while excluding the site main effect, irrelevant for entry ranking [60]. For sake of clarity, the graphs included just a subset of top-performing genotypes, namely, the two top-yielding RILs in each environment or across environments, and the four reference cultivars.

Interrelationships between traits were investigated by Pearson’s correlation analysis. All analyses of phenotypic data were carried out using SAS/STAT® software [62].

### DNA isolation, GBS library construction, and sequencing

DNA was extracted from green tissue that was collected from bulked stipules of four F_6_ plants per line using a CTAB method [63], checking its quality on 1% agarose gel. We adopted the GBS protocol by Elshire et al. [27] with modifications. After quantification by a Quant-iT™ PicoGreen® dsDNA assay kit (Life Technologies, P7589), each DNA sample (100 ng) was digested with *Ape*KI (NEB, R0643L) and then ligated to a unique barcoded adapter plus a common adapter. Equal volume of the ligated product was pooled and cleaned up with QIAquick PCR purification kit (QIAGEN, 28104) for subsequent amplification. In PCR, 50 ng template DNA was mixed with two primers and KAPA Library Amplification Readymix (KAPA Biosystems KK2611). Amplification was carried out on a thermocycler for 10 cycles with 10 s of denaturation at 98 °C, followed by 30 s of annealing at 65 °C and 30 s extension at 72 °C. Each library was sequenced in two lanes on Illumina HiSeq 2000 at the Genomic Sequencing and Analysis Facility of the University of Texas, Austin, TX.

### Genotype SNP calling and data filtering

GBS raw reads (100 bp, single end) were demultiplexed and trimmed for restriction enzyme remnants. The three parental lines (Attika, Isard and Kaspa) plus the two lines with highest number of reads were used to assemble a mock reference genome using the dDocent pipeline [64], which internally leverages the Rainbow tool [65]. For this step, we selected the similarity parameter *c* = 0.9. We used dDocent also for the successive steps of reads alignment and SNP calling, which internally use bwa [66] and freebayes [67], respectively. All parameters were kept at default values. The final genotype matrix, in the form of a vcf file, was further filtered for quality using the VCFtools software [68] with parameters *--minQ 30 --max-non-ref-af 1 --non-ref-af 0.001*. The resulting data set was filtered for increasing levels of allowed missing values, excluding markers whose missing rate over genotypes was greater than a fixed threshold of 1, 5, 10, 20, and 30%. Markers that were monomorphic or with minor allele frequency < 5% were removed. Following Nazzicari et al. [69], we estimated missing data by Random Forest imputation [70] using the R package MissForest [71] with the configuration ntree = 100, maxiter = 10, encoding genotypes as categorical data (factors).

### Genomic regression models and data configurations

We considered two GS models that stood out for predictive ability in a previous comparison of models for pea grain yield [13], namely, Ridge regression BLUP (rrBLUP) and Bayesian Lasso (BL). The rrBLUP model assumes a linear mixed additive model where each marker is assigned an effect as a solution of the equation [72]:

$$ Y=\mu +G\ u+\varepsilon $$

where *Y* is the vector of observed phenotypes, *μ* is the mean of *Y*, *G* is the genotype matrix (e.g., {0,1,2}), *u* ~ *N (0, Iσ*_*u*_^*2*^*)* is the vector of marker effects, and *ε ~ N (0, Iσ*_*e*_^*2*^*)* is the vector of residuals. Solving with the standard ridge-regression method, the solution is:

$$ \hat{u}=G\hbox{'}{\left(G\;G\hbox{'}+\lambda\;I\right)}^{-1}\;\left(Y-\mu \right) $$

where *λ = σ*_*e*_^*2*^ */ σ*_*u*_^*2*^ is the ridge parameter, representing the ratio between residual and markers variance. Given the vector of effects, it is possible to predict phenotypes and estimate genetic breeding values. The rrBLUP model assumes that the effects of all loci have a common variance, making it more suitable for traits influenced by a large number of minor genes. In contrast, Bayesian models assume relatively few markers with large effects, allowing different markers to have different effects and variances [73]. These models assign prior densities to markers effects, thereby inducing different types of shrinkage. The solution is obtained by sampling from the resulting posterior density. We selected the BL model as described in [74]. Details on the implementation of rrBLUP and BL models were the same as those reported in [13].

We also considered a third GS model, namely, Genomic BLUP (G-BLUP) [75], which stood out in another comparison of GS models for pea traits [30]. This model was described as mathematically equivalent to rrBLUP under certain conditions [76], but exploits genomic relationships between individuals to estimate breeding values, has reduced dimensions, and does not require thousands of iterations for its construction [73]. We estimated the genomic covariance matrix via a kinship matrix as described in [77].

We assessed the model predictive ability as Pearson’s correlation between observed and predicted phenotypes in a 10-fold stratified cross-validation scheme (where training and validation held 90 and 10% of data, respectively) for each regression model (rrBLUP, BL, G-BLUP) and possible genotype missing data threshold (1, 5, 10, 20, 30%). Here and in following analyses, predictive abilities were assessed separately for each RIL population and then averaged across populations (to avoid bias associated with different population mean value). Each cross-validation test was repeated 50 times, averaging the results to ensure numerical stability. Regression models, cross-validation and predictive ability estimations were all implemented using the R package GROAN [78]. We selected for subsequent analyses the model and data configuration that maximized the average predictive ability across all traits.

Population structure, which may improve the predictive ability of GS models [79], was taken into account by adding in the model a RIL population fixed factor as a 3 × *n* incidence matrix, where *n* is the number of samples. For the selected GS model and data configuration, we compared the predictive ability based on cross-validations obtained with and without imputed structure information. In addition, we verified the predictive ability of GS models trained on the single RIL populations (which implied less data for model construction). Results from these preliminary investigations supported the selection of Bayesian Lasso without structure information with 1% missing data threshold.

### Inter-environment and inter-population predictive abilities

We assessed the intra-population, inter-environment predictive ability of GS models, i.e., the model predictive ability for other environments than that of model construction using the same RIL populations for model training and selection. By turns, the selected GS model built in one (test) environment was used to predict in each of the other two (validation) environments the phenotypic values of independent lines selected by a 10-fold stratified cross-validation scheme with 50 repetitions, averaging the results across RIL populations.

Other GS model assessments regarded the inter-population predictive ability, i.e., the ability of one model constructed from phenotyping data of one RIL population to predict data of the other RIL populations. This was envisaged for predictions relative to data of the same environment used for model construction (inter-population, intra-environment prediction) and for predictions relative to each of the other two independent environments (inter-population, inter-environment prediction). We used by turns each environment and RIL population for model training (using all data from the relevant RIL population), averaging the results across the single training environments.

### Comparison of genomic vs phenotypic selection

The phenotypic correlation of the phenotypic data in one (validation) environment with either the phenotypic data averaged across the other two (test) environments or the GS-based breeding values obtained from the same data, averaging the results across all possible environment combinations and RIL populations, provided a preliminary assessment of phenotypic vs genome-enabled predictions. This comparison aimed to assess the possible loss (or gain) of predictive ability derived from GS modelling of phenotypic data relative to that of phenotypic data themselves.

Further analyses were carried out to compare GS vs PS in terms of selection efficiency for future selection activities, taking into account their possible differences for length of selection cycle and selection costs. For GS, we hypothesized two environments to be used for model building, i.e., the model training on line mean values across two environments (rather than one, as in the earlier assessment of inter-population predictions), as reasonable in the presence of sizeable GEI, and left one environment for model validation. Also here, the three environments acted by turns as test or validation. GS gains were assessed for two scenarios. The former scenario implied model training and predictions for the same RIL population (intra-population, inter-environment predictions), estimating the predictive ability (*r*_*Ab*_) using 90% of the RIL data for model construction and 10% for validation, with a cross-validation procedure repeated 50 times. The latter scenario contemplated the GS model training based on all data of one RIL population for prediction of the other two RIL populations (inter-population, inter-environment predictions). In both cases, the *r*_*Ab*_ values averaged across RIL populations and all possible sets of training environments were used to estimate GS model accuracy (*r*_*Ac*_) values according to [33] as: *r*_*Ac*_ = *r*_*Ab*_ / *H*, where *H* is the square root of the broad-sense heritability on a genotype mean basis in the validation environment estimated as described earlier. The mean value of *r*_*Ac*_ across RIL populations and validation cycles was inputed in the following formula for estimation of the expected genetic gain per selection cycle from GS [39]:

$$ {\Delta \mathrm{G}}_G={i}_G\ {r}_{Ac}\ {s}_A $$

where *i*_*G*_ = standardized selection differential for GS, and *s*_*A*_ = standard deviation of breeding values. We computed the expected genetic gain per year as:

$$ {\varDelta G}_G'=\left({i}_G\ {r}_{Ac}\ {s}_A\right)/{t}_G $$

where *t*_*G*_ = duration in years of one GS cycle, which was set to 0.5 under the hypothesis of two possible selection cycles per year for GS (one off-season and one ordinary).

The expected genetic gain per year from PS is [80]:

$$ {\varDelta G}_P'=\left({i}_P\ H\ {s}_A\ \right)/{t}_P $$

where *i*_*P*_ = standardized selection differential for PS, *t*_*P*_ = duration in years of one PS cycle, and *H* = square root of the broad-sense heritability on a genotype mean basis across the experiments hypothesized for selection. We hypothesized two selection experiments, each with three replications, accommodated either at two sites in the same year (implying *t*_*P*_ = 1) or in two years at the same site (implying *t*_*P*_ = 2). For each RIL population, we estimated the broad-sense heritability on a genotype mean basis across each of the three possible pairs of environments from the relevant *S*_*G*_^*2*^, *S*_*GE*_^*2*^ and *S*_*e*_^*2*^ components of variance as described earlier. The mean *H* value averaged across the three RIL populations and the three pairs of environments was used for predicting gains from PS.

From the formulae above, a comparison of GS vs PS in terms of predicted genetic gain per year for same overall costs equates to comparing (*i*_*G*_ *r*_*Ac*_ / *t*_*G*_) vs (*i*_*P*_ *H* / *t*_*P*_), considering the impact on *i*_*G*_ and *i*_*P*_ values of different evaluation cost per genotype of GS and PS. For the hypothesized selection scenarios, we estimated a cost of € 200 for both PS scenarios (given the same number of selection environments), and € 40 for GS [28]. For a fixed budget available, this implied five-fold more evaluated genotypes and, hence, five-fold lower selected fraction for GS relative to PS. For large genotype numbers, the ratio of *i*_*G*_ to *i*_*P*_ is 1.379 (2.421/1.755) when considering selected fractions of 2% for GS and 10% for PS, and 1.539 (2.154/1.400) when considering selected fractions of 4% for GS and 20% for PS [80]. We adopted the intermediate ratio value within this range, setting *i*_*G*_ = 1.46 *i*_*P*_. Therefore, the relative efficiency of GS vs PS in terms of predicted genetic gain per year for same budget coincided with the ratio of (1.46 *r*_*Ac*_ / *t*_*G*_) to (*H* / *t*_*P*_), setting *t*_*G*_ = 0.5 and envisaging the values 1 or 2 for *t*_*P*_.

### Genome-wide association study

A genome-wide association study (GWAS) was carried out for each trait of the three RIL populations using the *egscore* function in the R package GenABEL [81]. The main aim of this study was to provide preliminary information on the trait genetic control by exploring the number of independent genomic areas that displayed linkage with the target traits. Data were considered stratified, with each population acting as a stratum. Therefore, the association scores were computed within each stratum and then combined. Principal components analysis was adopted to take population structure into account according to [82], correcting for inflation (*Pc1df* in GenABEL nomenclature). The resulting scores were further verified for inflation rate using Benjamini and Yekutieli’s False Discovery Rate criterion [83]. A marker ranking per trait was obtained, setting an association score threshold of three (corresponding to *P* < 0.001 significance of the association). Linkage disequilibrium (LD) was measured between the significantly associated markers and those used for a consensus map reported in [42], where trait-marker associations were studied on a germplasm set that included 180 lines in common with the current study. The squared correlation coefficient (*r*^*2*^) was used as a measure of LD, considering as highly linked the markers showing *r*^*2*^ > 0.8).

## Additional files

Additional file 1: **Table S1.** Climate and soil characteristics of three pea test environments, and long-term climate characteristics of the test sites. (DOCX 12 kb)

Additional file 2: **Table S2.** Mean value of the parent lines of three connected RIL populations. (DOCX 14 kb)

Additional file 3: **Figure S1.** Number of polymorphic markers available in three pea RIL populations for five genotype missing data thresholds. (TIF 136 kb)

Additional file 4: **Table S3.** Intra-population predictive ability (PA) of the best-performing genomic selection model for each specific trait (GS-ST) and the best-performing genomic selection model across traits (GS-AT). Values averaged across two (lodging susceptibility) or three (other traits) pea RIL populations. **Table S3bis.** Root Mean Square Difference (RMSD) between intra-population predictive ability (PA) of each combination of genomic selection models and missing data thresholds (MDT) versus the best-performing model for each specific trait. Values averaged across 13 traits reported in Table S3. Table sorted by ascending RMSD, with best-performing models on top. (DOC 56 kb)

Additional file 5: **Table S4.** Rank, association score and sequence of markers associated with onset of flowering (OF), grain yield (GY), lodging susceptibility (LS), seed weight (SW) and winter survival (WS), and linkage disequilibrium analysis of these markers with SNP array markers in the consensus map by Ferrari et al. [42]. (XLS 44 kb)

Additional file 6: **Archive S1.** A compressed archive containing the reference genome, the genotypes for five missing data thresholds, the kinship matrices, the phenotypes and a phenotype-genotype coupling table. (ZIP 8471 kb)

## Data Availability

A compressed archive containing the reference genome, the genotypes for five missing data thresholds, the kinship matrices, the phenotypes and a phenotype-genotype coupling table is provided in Additional file 6: Archive S1.

## Abbreviations

AMMI: Additive main effects and multiplicative interaction
ANOVA: Analysis of variance
BL: Bayesian Lasso
BLUP: best linear unbiased prediction
G-BLUP: genomic BLUP
GBS: Genotype-by-sequencing
GEI: Genotype × environment interaction
GS: Genomic selection
GWAS: Genome-wide association study
PC: Principal component
PS: Phenotypic selection
RIL: Recombinant inbred line
rrBLUP: Ridge regression best linear unbiased prediction
SNP: Single nucleotide polymorphism
