# Supplementary material for: Pea genomic selection for Italian environments
Source: BMC Genomics. 2019 Jul 22;20:603. doi: 10.1186/s12864-019-5920-x (PMC6647272; doi:10.1186/s12864-019-5920-x)
Supplement: Supplementary file 1 — Table S1. Climate and soil characteristics of three pea test environments, and long-term climate characteristics of the test sites. (DOCX 12 kb) [file 12864_2019_5920_MOESM1_ESM.docx]

**Table S1** Climate and soil characteristics of three pea test environments, and long-term climate characteristics of the test sites

| Item | Lodi 2013-14 | Lodi 2014-15 | Perugia 2013-14 | Lodi long-term | Perugia long-term |
| --- | --- | --- | --- | --- | --- |
| Crop management system | Organic | Conventional | Organic | - | - |
| Rainfall, Jan.-Mar. (mm) | 343 | 198 | 280 | 161 | 177 |
| Rainfall, Apr.-May (mm) | 122 | 147 | 179 | 154 | 142 |
| Absolute minimum daily temp. (°C) | –5.7 | –11.6 | –3.6 | –7.7 | –5.0 |
| Mean of max. daily temp., May (°C) | 23.2 | 23.9 | 23.4 | 21.8 | 23.0 |
| Soil texture^a^ | Silt-loam | Sandy-loam | Silty-clay-loam | - | - |
| Soil pH | 7.9 | 6.3 | 7.6 | - | - |

^a^ According to FAO (2006) Guidelines for soil description, 4th. Rome: Food and Agricultural Organization.
