# Supplementary material for: Pea genomic selection for Italian environments
Source: BMC Genomics. 2019 Jul 22;20:603. doi: 10.1186/s12864-019-5920-x (PMC6647272; doi:10.1186/s12864-019-5920-x)
Supplement: Supplementary file 2 — Table S2. Mean value of the parent lines of three connected RIL populations. (DOCX 14 kb) [file 12864_2019_5920_MOESM2_ESM.docx]

**Table S2** Mean value of the parent lines of three connected RIL populations

|  |  |  | | Mean value^c^ | |  | |
| --- | --- | --- | --- | --- | --- | --- | --- |
| Trait^a^ | Environment^b^ | Attika | | Isard | | Kaspa | |
| GY (t/ha) | Lo14 | 4.881 | c | 6.356 | ab | 7.198 | a |
| GY (t/ha) | Lo15 | 1.600 | b | 6.690 | a | 2.335 | b |
| GY (t/ha) | Pg14 | 2.129 | b | 2.594 | ab | 3.344 | a |
| OF (dd from Apr. 1) | Lo14 | 10.22 | b | 10.89 | b | 17.67 | a |
| OF (dd from Apr. 1) | Lo15 | 14.47 | b | 9.70 | c | 25.37 | a |
| OF (dd from Apr. 1) | Pg14 | 11.22 | b | 11.33 | b | 18.67 | a |
| LS (score 1=min, 5=max) | Lo14 | 1.56 | b | 1.89 | b | 3.22 | a |
| LS (score 1=min, 5=max) | Lo15 | 3.53 | a | 3.88 | a | 3.58 | a |
| LS (score 1=min, 5=max) | Pg14 | 2.89 | a | 1.44 | b | 2.33 | a |
| SW (g) | Lo14 | 0.245 | a | 0.188 | c | 0.214 | b |
| SW (g) | Lo15 | 0.199 | a | 0.169 | c | 0.191 | b |
| SW (g) | Pg14 | 0.207 | a | 0.168 | b | 0.205 | a |
| WS (proportion) | Lo15 | 0.660 | b | 0.900 | a | 0.483 | c |

^a^ GY, grain yield; OF, onset of flowering; LS, lodging susceptibility; SW, individual seed weight; WS, winter survival.

^b^ Lo14, Lodi 2013-14; Lo15, Lodi 2014-15; Pg14, Perugia 2013-14.

^c^ Row means followed by different letter differ at *P* < 0.05.
