# Supplementary figures and images for: Pea genomic selection for Italian environments
Source: BMC Genomics. 2019 Jul 22;20:603. doi: 10.1186/s12864-019-5920-x (PMC6647272; doi:10.1186/s12864-019-5920-x)

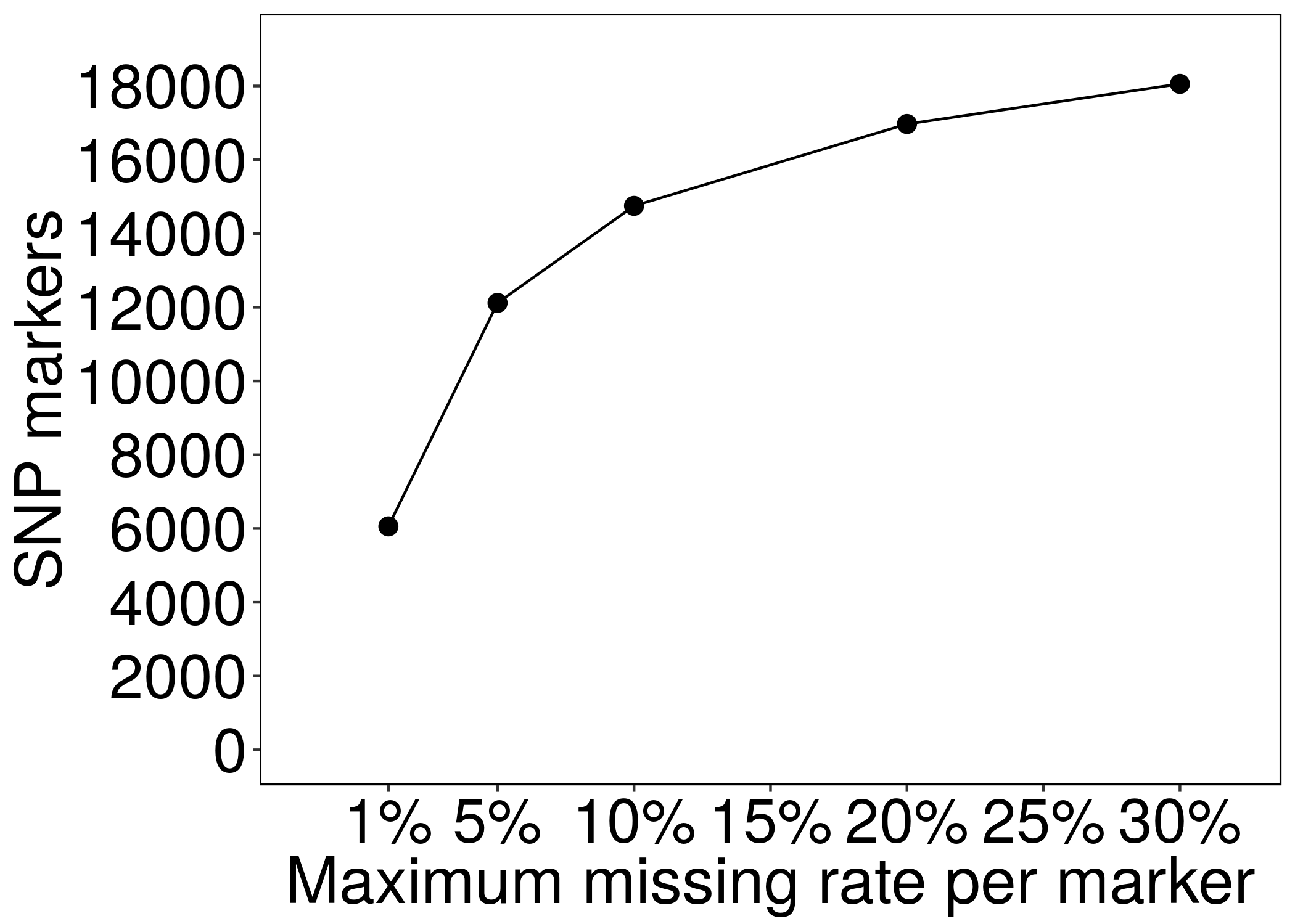

Supplement: Supplementary file 3 — Figure S1. Number of polymorphic markers available in three pea RIL populations for five genotype missing data thresholds. (TIF 136 kb) [file 12864_2019_5920_MOESM3_ESM.tif]
