# Supplementary material for: Pea genomic selection for Italian environments
Source: BMC Genomics. 2019 Jul 22;20:603. doi: 10.1186/s12864-019-5920-x (PMC6647272; doi:10.1186/s12864-019-5920-x)
Supplement: Supplementary file 4 — Table S3. Intra-population predictive ability (PA) of the best-performing genomic selection model for each specific trait (GS-ST) and the best-performing genomic selection model across traits (GS-AT). Values averaged across two (lodging susceptibility) or three (other traits) pea RIL populations. Table S3bis. Root Mean Square Difference (RMSD) between intra-population predictive ability (PA) of each combination of genomic selection models and missing data thresholds (MDT) versus the best-performing model for each specific trait. Values averaged across 13 traits reported in Table S3. Table sorted by ascending RMSD, with best-performing models on top. (DOC 56 kb) [file 12864_2019_5920_MOESM4_ESM.doc]

**Table S3** Intra-population predictive ability (PA) of the best-performing genomic selection model for each specific trait (GS-ST) and the best-performing genomic selection model across traits (GS-AT). Values averaged across two (lodging susceptibility) or three (other traits) pea RIL populations

|  |  | GS-ST | |  | GS-AT^d^ |
| --- | --- | --- | --- | --- | --- |
| Trait^a^ | Environment^b^ | PA | Description^c^ |  | PA |
| GY | Lo14 | 0.484 | G-BLUP, MDT = 20 % |  | 0.460 |
| GY | Lo15 | 0.707 | BL, MDT = 1 % |  | 0.707 |
| GY | Pg14 | 0.313 | BL, MDT = 1 % |  | 0.313 |
| OF | Lo14 | 0.770 | BL, MDT = 10 % |  | 0.751 |
| OF | Lo15 | 0.714 | rrBLUP, MDT = 30 % |  | 0.704 |
| OF | Pg14 | 0.806 | BL, MDT = 10 % |  | 0.778 |
| LS | Lo14 | 0.232 | BL, MDT = 10 % |  | 0.215 |
| LS | Lo15 | 0.598 | BL, MDT = 10 % |  | 0.584 |
| LS | Pg14 | 0.442 | BL, MDT = 5 % |  | 0.416 |
| SW | Lo14 | 0.779 | BL, MDT = 20 % |  | 0.750 |
| SW | Lo15 | 0.653 | BL, MDT = 10 % |  | 0.607 |
| SW | Pg14 | 0.727 | BL, MDT = 10 % |  | 0.718 |
| WS | Lo15 | 0.561 | BL, MDT = 5 % |  | 0.549 |

^a^ GY, grain yield; OF, onset of flowering; LS, lodging susceptibility; SW, individual seed weight; WS, winter survival.

^b^ Lo14, Lodi 2013-14; Lo15, Lodi 2014-15; Pg14, Perugia 2013-14.

^c^ BL, Bayesian Lasso; rrBLUP, Ridge regression BLUP; G-BLUP, Genomic BLUP; MDT, genotype missing data thresholds (from 1 % to 30 %).

^d^ BL with MDT = 1 %.

**Table S3bis** Root Mean Square Difference (RMSD) between intra-population predictive ability (PA) of each combination of genomic selection models and missing data thresholds (MDT) *versus* the best-performing model for each specific trait. Values averaged across 13 traits reported in Table S3. Table sorted by ascending RMSD, with best-performing models on top.

| Model | MDT | RMSD |
| --- | --- | --- |
| BL | 1 % | 0.0218045 |
| BL | 5 % | 0.0219192 |
| BL | 20 % | 0.0222769 |
| BL | 30 % | 0.0234431 |
| BL | 10 % | 0.0248726 |
| rrBLUP | 20 % | 0.0255035 |
| rrBLUP | 5 % | 0.0257295 |
| rrBLUP | 30 % | 0.0257686 |
| rrBLUP | 10 % | 0.0267740 |
| rrBLUP | 1 % | 0.0343288 |
| G-BLUP | 20 % | 0.0348132 |
| G-BLUP | 30 % | 0.0353981 |
| G-BLUP | 10 % | 0.0370404 |
| G-BLUP | 5 % | 0.0421331 |
| G-BLUP | 1 % | 0.0536379 |
